# Extending Long‐Term Avian Studies Alters Temporal and Climate‐Driven Trend Conclusions

**DOI:** 10.1002/ece3.71878

**Published:** 2025-07-28

**Authors:** Irene Zanandrea, Juan Moreno, Alejandro Cantarero

**Affiliations:** ^1^ Natural History Museum University of Oslo Oslo Norway; ^2^ Department of Evolutionary Ecology National Museum of Natural Sciences, CSIC Madrid Spain; ^3^ Department of Physiology, Veterinary School Complutense University of Madrid Madrid Spain

**Keywords:** climate change, conservation strategies, long‐term effects, long‐term population studies, pied flycatcher, reproduction

## Abstract

Long‐term population studies are crucial for understanding the impacts of climate change on biodiversity; however, predictions based on short‐term data may be unreliable. Here, we analyse yearly averages of reproductive parameters (laying date, nestling condition, and female condition) of a Pied Flycatcher ( *Ficedula hypoleuca* ) population in central Spain across one, two, and three decades of study to evaluate how study duration affects conclusions about population trends. Our findings reveal that initial trends deduced from studies of shorter duration often weaken, invert, or disappear when extended durations are included. For example, advancements in laying dates only become apparent after more than two decades, while trends in nestling and female condition vanish over time. Additionally, we observed unexpected responses, such as delays in laying during exceptionally hot prelaying periods. These results emphasize the necessity of long‐term studies spanning multiple generations to accurately assess the impacts of climate change and inform effective conservation strategies. Our work underscores the risks of relying on short‐term data to predict long‐term ecological trends.

## Graphical abstract

Long‐term population studies are essential for understanding the effects of climate change on biodiversity, yet predictions based on short‐term data may be unreliable. Our findings reveal that initial trends derived from shorter studies often weaken, invert, or disappear when extended data are included.

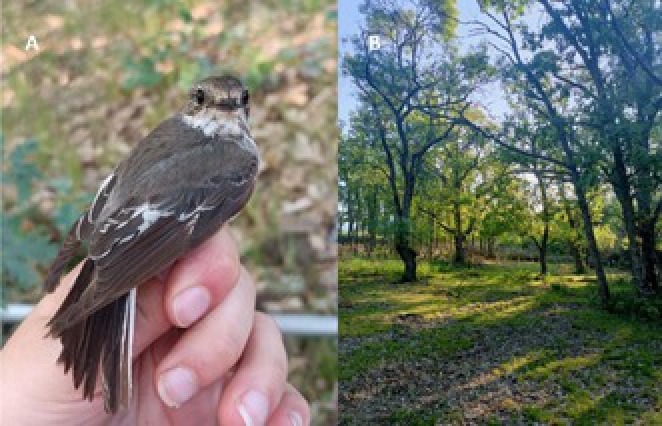

## 1 Introduction

In a scenario of climate change, the interest in exploring temporal trends in population parameters has led, during recent decades, to an exponential increase in these long‐term population studies spanning a wide range of organisms to predict future evolutionary trends (Radchuk et al. 2019; Shipley et al. 2020; Smart et al. 2021; Mainwaring et al. 2021). However, the pressure to detect and identify future scenarios for a range of populations has led to a rush to publish identifiable trends as soon as studies have lasted reasonably long. Studies including a few generations have been published for short‐lived organisms, leading to predictions based on IPCC scenarios for future climate trends (Walther et al. 2002; Sunday et al. 2012; Parmesan 2006). However, there is the risk that predictive scenarios may be based on conclusions sustained by insufficient information for some populations, given the possibility of short‐term responses to chaotic weather fluctuations being incorporated in studies of brief duration (Hughes et al. 2017; Liu et al. 2021). As the duration of long‐term population studies increases continuously, the possibility of checking the accuracy of scenarios based on shorter‐term approaches can be easily checked by comparing trends based on data sets of increasing length (Tomotani et al. 2018; Selonen et al. 2021; Cadahía et al. 2017; Both et al. 2006; Visser et al. 2015; Le Vaillant et al. 2021).

Among the most well‐studied organisms in climate change research, birds occupy a central position given the availability of long‐term data sets based on almost a century of population studies (Grant and Grant 2002; Lee 2021; Radchuk et al. 2019; Shipley et al. 2020). Many studies have shown that climate change is profoundly impacting the breeding phenology of birds (Radchuk et al. 2019; Shipley et al. 2020; Smart et al. 2021; Mainwaring et al. 2021). Although species have presumably always responded to climatic fluctuations throughout their evolutionary history, a primary concern arises due to the rapid rate of climate change presently experienced (Root et al. 2003; Lee 2021).

Many long‐term avian population studies are based on cavity‐nesting passerines given the relative simplicity of gathering data from nest‐box breeding birds (González‐Braojos et al. 2017; Sanz et al. 2003; Lambrechts et al. 2010). The widespread use of artificial nestboxes has led to significant advances in our knowledge of the ecology, behavior, and physiology of cavity‐nesting birds, especially small passerines (Lambrechts et al. 2010). Thanks to long‐term monitoring data sets of marked individuals, it is nowadays possible to estimate life‐history and demographic parameters (Camacho et al. 2017; Culina et al. 2021). Long‐term studies on wild populations, with individually known individuals, play an essential role in detecting and understanding the temporal trends in life‐history traits and in estimating the heritability and selection pressures on life‐history traits (Visser 2008). Some of these studies have been conducted for a sufficient number of years to allow comparisons of trends based on an increasing duration of data collection.

Here, we use standard information on reproductive parameters of a nest‐box breeding population of pied flycatchers ( *Ficedula hypoleuca* ) from central Spain to evaluate whether conclusions about trends based on data from one, two, and three decades are coherent. Three scenarios can be predicted: either trends in reproductive parameters based on short‐term studies are confirmed by prolonging study duration, or they are contradicted by adding further years (inverse tendencies), or, finally, they disappear.

## 2 Materials and Methods

### 2.1 Study Species

The pied flycatcher is a small (12 g), insectivorous, long‐distance migratory passerine of European woodlands (Lundberg and Alatalo 1992). It is a secondary‐cavity nester that breeds in secondary cavities across Europe's temperate forests (Burgess et al. 2018), and readily occupies human‐provided nestboxes (Nater et al. 2023), thereby facilitating comparative and experimental field investigations (Lambrechts et al. 2012). Pied flycatchers are short‐lived (on average just a few years) and migrate annually between boreal/temperate breeding grounds in Europe and wintering areas in western Africa (Nater et al. 2023). Spain harbors the southernmost breeding populations of this species: there, pied flycatchers arrive in mid‐April and females start breeding in early May (Sanz et al. 2003). After nest‐building, the female lays one egg per day, incubates, and broods the young alone, although she receives food from her mate during laying and incubation (Cantarero et al. 2014; Moreno et al. 2011). Both parents feed the nestlings, which fledge within 16–20 days after hatching (Moreno 2020, 2022).

### 2.2 Fieldwork and Study Area

**FIGURE 1:**
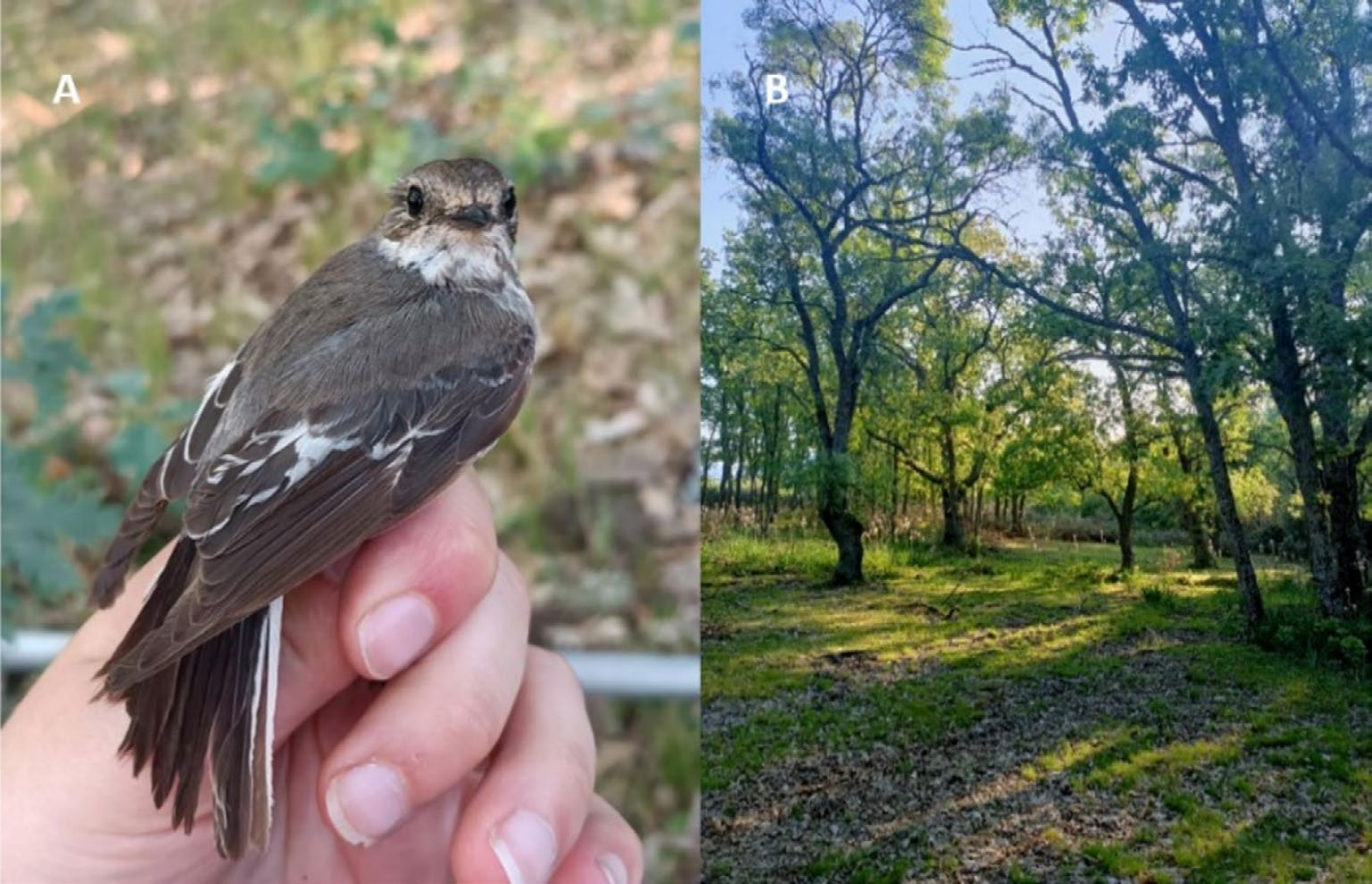
Study species and study area. (A) A female individual of the pied flycatcher from the studied population. (B) The study area is in Valsaín, central Spain.

All the breeding data were collected from a population of *Ficedula hypoleuca iberiae* (Figure 1A) breeding in nest‐boxes in a deciduous montane oak forest (*Quercus pyrenaica*) at 1200 m a.s.l. in Valsaín, Segovia, central Spain (40°54′ N, 04°01′ W) (Figure 1B) between 1991 and 2022. In this study site, 300 nestboxes have been installed and placed hanging from a branch attached to a metal hook (see Lambrechts et al. (2010) for dimensions, structure, and placement of nest boxes). As described by González‐Braojos et al. (2017), all nest‐boxes (covering an area of roughly 45 ha) were inspected repeatedly each spring, and occupation by pied flycatchers was recorded. Adults and nestlings were ringed, and the following measurements were obtained: tarsus length was measured with a digital caliper to the nearest 0.01 mm, and mass was measured with a digital balance with a precision of 0.1 g (González‐Braojos et al. 2017). Nestlings were ringed at 13 days of age (hatching day = day 1, range 12–14 days).

### 2.3 Data‐Sets

In this research, a long‐term (1991–2022) individual‐based data‐set was used to explore the hypothesis that extending long‐term avian population studies can lead to revised conclusions regarding temporal and climate‐driven trends, as the same population was previously included in studies of shorter duration (Sanz et al. 2003; González‐Braojos et al. 2017). To do so, the analysis started with two different data‐sets, one with the breeding information of the studied population (individual‐based data‐set) and the other with the local climate of the study area (climatic data‐set).

#### 2.3.1 Breeding Data‐Set

The breeding data‐set is a thirty‐one‐year data‐set in which various breeding and morphological features have been recorded yearly per nest. Nest‐based data include breeding dates, breeding success, and individual morphological characteristics. As for the breeding dates, for each pair (a male and a female) of a given nest, a list of important parameters was registered: the laying date (LD), the clutch size (CS), the hatching date (HD) and the brood size 3 and 13 days after hatching (BS3 and BS13). As for the individual morphological traits, for both the pairs and the nestlings, both the individual ID (when possible) and other biometric values, namely mass (±0.1 g), length of the tarsus (±0.01 mm) and length of the wing (±0.1 mm), were recorded. Averages were obtained for broods in the case of nestling condition.

#### 2.3.2 Climatic Data‐Set and Local Climate

All the local climatic information was provided by the Spanish Meteorological Agency (AEMET 2021). The weather station (Segovia, number station: 2465, coordinates: 40°56′43″ N, 4°7′35″ W) is located 9 km from the study area at the same altitude (González‐Braojos et al. 2017). From the AEMET website (2021), the daily weather variables were extracted for every year of the study period (1991–2022). Therefore, a 31‐year climatic data set was created to contain daily climatic variables from which only temperature and rainfall patterns (precipitation) were retained.

#### 2.3.3 Combined Data‐Set for Population Trends: Full and Reduced Dataset

The two data‐sets (breeding and climate) were combined to provide yearly nest‐specific information regarding the effect of weather conditions on reproductive patterns during three reproductive phases: prelaying, incubation, and nestling periods. For these three phases, the climatic variables were calculated as follows: for the prelaying data, it was considered the period from 10 days before the laying date (LD, date of first egg) to 3 days before it, when the first egg is formed (Slagsvold and Lifjeld 1989). For the incubation period, data from 12 days before the hatching date (HD, date when the first nestling was detected during daily inspections) were considered (the minimum incubation time found in the population). Finally, for the nestling period, the time considered spanned between the hatching date and the date when nestlings were 13 days old (12 days after HD, when nestlings complete their skeletal growth). Additionally, an index of body condition for chicks, males, and females was estimated. This was obtained by dividing body mass by wing length raised to the third power (mass/(wing length)^3^) ratio (Pravosudov et al. 1999). From the full combined data‐set, we derived a reduced data‐set by calculating the yearly average of each measurement. This resulted in obtaining a single data point for each year (reduced data‐set) for both breeding and climate variables. This approach was implemented to address the issue of pseudoreplicates and correct for an excess of statistical power relative to the amount of raw data contained in the complete data‐set. As we are analyzing yearly trends, all data points from the same year share common conditions that make them not fully statistically independent. However, it is important to acknowledge that relying on only one data point per year significantly reduces the statistical power of our analyses.

### 2.4 Statistical Analyses

We wanted to assess whether prolonging the duration of avian population studies can result in altered conclusions regarding trends influenced by time (temporal) and climate. To do so, we computed phenological trends using the yearly averages by considering three different study durations: (1) from 1991 to 2000, (2) from 1991 to 2010, and (3) from 1991 to 2022. To understand both the yearly trends and the climatic associations of climate with breeding variables (for temperature and precipitation independently), we tested both linear and non‐linear (quadratic) relationships. All statistical analyses were performed using R software (version 4.2.3, R Foundation for Statistical Computing) and RStudio; linear regression models (LMs) and quadratic regression models were performed. We assessed the relative strength of linear and quadratic associations through their coefficients of determination (R^2^). Models with R^2^ lower than 0.01 will not be discussed. The choice of the best model for each dependent variable is justified by a formal model comparison based on Akaike's information criterion (AIC) performed using the package “AICcmodavg” in R.

#### 2.4.1 Temporal Trend (Time‐Breeding)

To investigate temporal trends in phenological and reproductive parameters, the following variables were chosen: laying date (LD), chick condition (Cc), and female condition (Fc). The laying date is pivotal since it contains information about the timing of reproduction: timing is crucial for chicks' survival since phenology is linked to food availability (Sanz et al. 2003). In addition, chick condition at fledging reflects offspring health and the potential ability to survive to the following year (Moreno et al. 2005). This parameter is used as a proxy for reproductive success. Female condition is related to adult reproductive costs and survival probabilities and may be related to population persistence (Garamszegi et al. 2004). In these analyses, we computed temporal trends considering only the variable “year” We calculated LMs and, secondly, quadratic models to determine which better explained the temporal patterns for each breeding parameter.

#### 2.4.2 Climatic Trend (Climate‐Breeding)

For every given reproductive variable (laying date, chicks' condition, and female condition), we evaluated the independent associations with temperature and precipitation across the three study durations (one, two, and three decades). These models were designed to capture the specific climate conditions potentially influencing the variables: prelaying climate conditions for LD, incubation climate conditions for female condition, and nestling climate conditions for chicks' condition. Like in the temporal analysis, we computed both Linear Regression models and Quadratic Regression models to estimate their relative strength.

## 3 Results and Discussion

In total, we collected information on 2753 nests during the 3‐decade period. Sample sizes for nests increased from 1076 for the first decade to 1929 for the first two decades and to 2753 for the complete duration of the study.

### 3.1 Laying Date

#### 3.1.1 Temporal Trends (Year‐Breeding Date)

**FIGURE 2:**
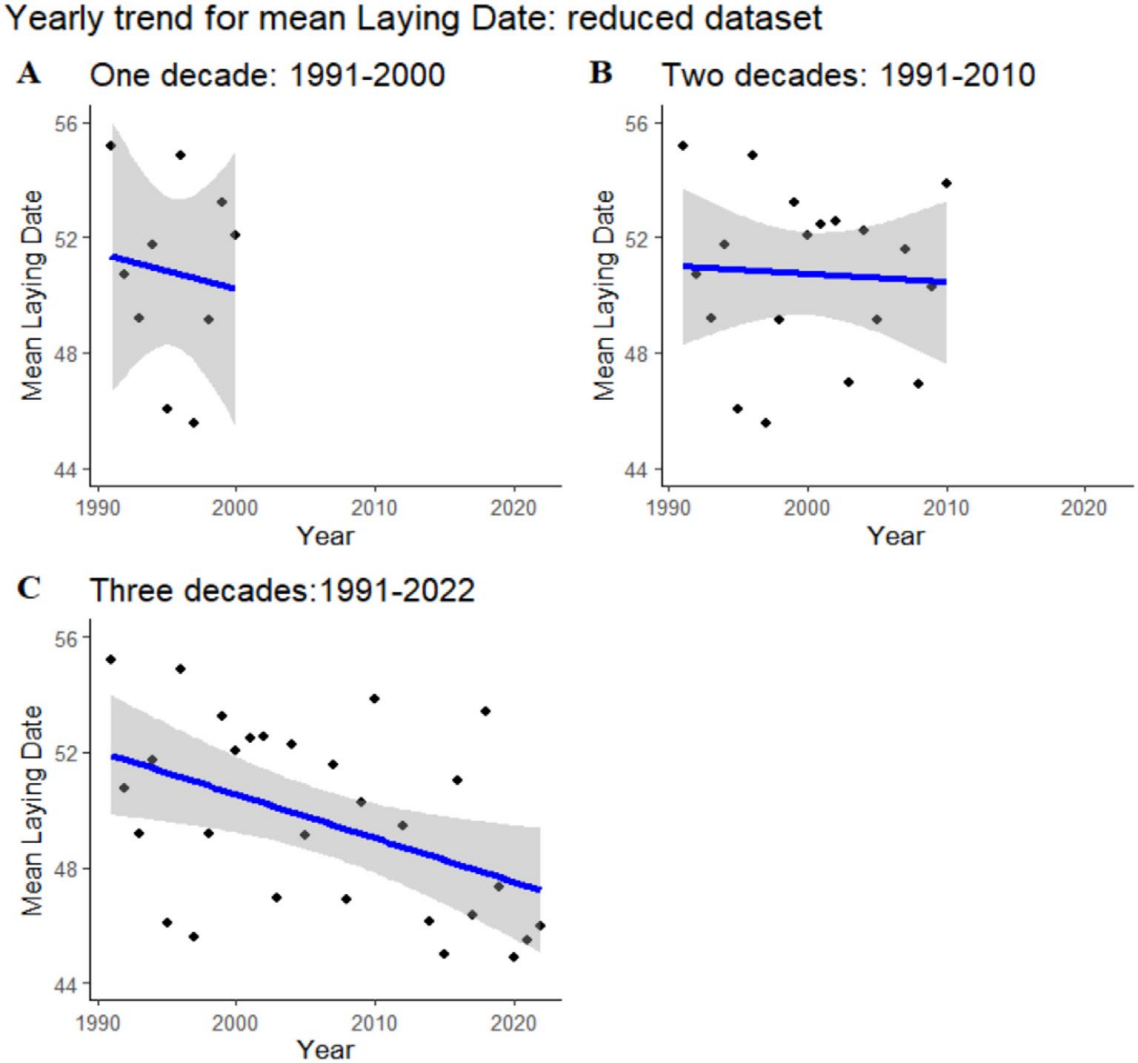
Temporal linear trends for temporal variation in laying date for the three study durations. (A) 1991–2000 (linear regression: *Y* = 301.01–0.13 x; *p* = 0.75), (B) 1991–2010 (linear regression: *Y* = 229.51–0.09 x; *p* = 0.49), and (C) 1991–2022 (linear regression: *Y* = 328.7–0.14 x; *p* = 0.05).

We performed linear and quadratic annual analyses to detect trends in laying dates. Based on the AIC model comparison, the linear models better explained the temporal trends, so we discuss only those. While the linear function was non‐significant for both the one‐decade period and the two‐decade period, it became significant when considering the full three‐decade period (Table 1, Figure 2). This analysis showed a trend towards earlier laying dates over the years. This indicates that prolonging the study duration enhances the ability to detect a trend.

**TABLE 1:** Linear model (LD ~ Year) for 1991–2022.

Coefficients	Estimate	Std. error	*t* value	pr(> |*t*|)
Intercept	328.70014	138.77659	2.369	0.0245
Year	−0.13918	0.006916	−2.012	0.0532

Temporal trends in avian laying date have been carefully analyzed in the context of environmental fluctuations since the beginning of long‐term population studies (Both and Visser 2001; Both et al. 2006; Sanz et al. 2003). Laying date variation has been related to changes in food supply, interspecific competition, and importantly, climatic fluctuations (Charmantier and Gienapp 2014). As breeding phenology should be adjusted to seasonal fluctuations in food availability, variation in breeding date may give information about possible maladjustments between avian populations and their resource supply (Both and Visser 2001; Sanz et al. 2003, Dunn et al. 2011; Hušek et al. 2013). Later, when the impacts of human‐induced climate change became one of the main concerns of population ecologists, variation in phenology began to be considered a crucial indication of population responses (Radchuk et al. 2019; Shipley et al. 2020; Smart et al. 2021; Mainwaring et al. 2021). If spring conditions were attained earlier in temperate areas, as shown with climatic models and actual field data (Burger et al. 2012), we should expect an advancement in breeding dates. This has been found in several populations across temperate breeding areas (Both and Visser 2001; Sanz et al. 2003; Both et al. 2006; Dunn and Winkler 1999; Visser and Gienapp 2019; González‐Braojos et al. 2017). However, differences in temporal adjustment have been detected between resident and migrating species (Travers et al. 2015; Newson et al. 2016; Søraker et al. 2022), with migrants responding less consistently to temporal trends in temperature and precipitation (Søraker et al. 2022). Migratory species partly depend on conditions in wintering areas to determine their migration schedule, so they have difficulties in responding to conditions in far‐away breeding areas (Both and Visser 2001). The long‐distance migratory pied flycatcher has been found to respond to changing climatic conditions across its Palearctic breeding range in an inconsistent manner, with some populations advancing their phenology, and others apparently resisting such changes despite potential declines in their reproductive outcomes (Søraker et al. 2022). Some conclusions may have been based on studies of too short a duration to ascertain real population responses (Sanz et al. 2003). Here, we have shown that it is only after more than two decades of study that advancements in breeding dates start to appear.

#### 3.1.2 Climatic Trends (Climate‐Breeding Date)

**FIGURE 3:**
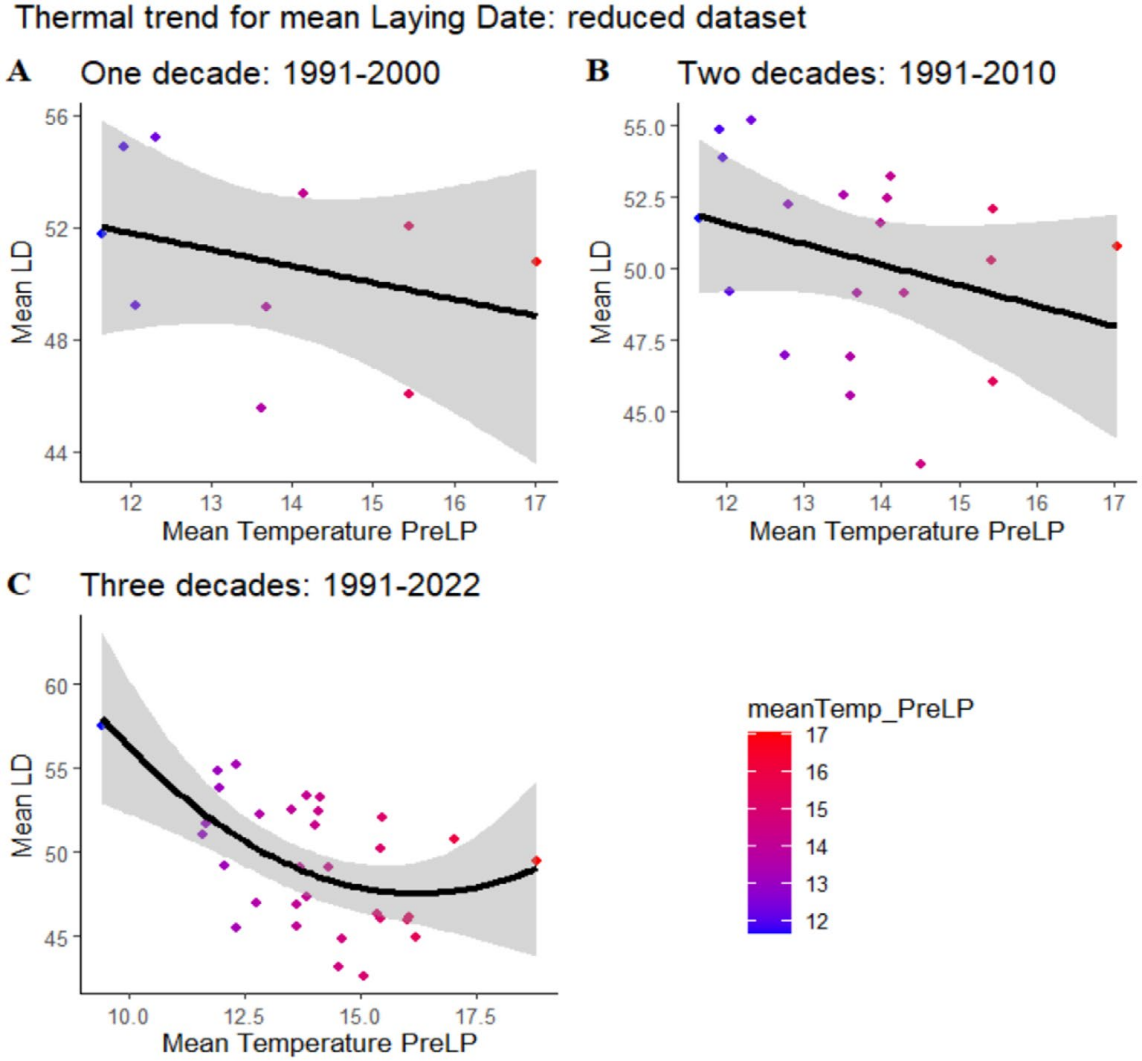
Thermal trend for laying date for the different decades. (A) 1991–2000 (linear regression: *Y* = 58.9–0.59 x; *p* = 0.36), (B) 1991–2010 (linear regression: *Y* = 60.2–0.72 x; *p* = 0.18), and (C) 1991–2022 (quadratic regression: *Y* = 107.27–7.37 x1 + 0.23 x2; *p* = 0.003).

The model selection based on AIC revealed a shift in which model better explains the thermal influence on laying date, depending on the number of decades included. For the first decade, the linear model was the best fit, and it remained so also when adding a second decade. However, when considering the entire study period (1991–2022), the quadratic model provided a better description. The first two linear models are non‐significant. Instead, the quadratic thermal model for the entire study period shows a significantly negative significant curvilinear relationship between the average pre‐laying temperature and laying date (Table 2). This indicates that with warmer temperatures, breeding starts earlier, although the negative tendency weakens for the highest temperatures (Figure 3). Again, prolonging the study led to the detection of a trend that was not there for shorter durations. Overall, we found only non‐significant trends for prelaying precipitation affecting laying date.

**TABLE 2:** Quadratic thermal model (LD ~ Temp2) for 1991–2022.

Coefficients	Estimate	Std. error	*T* value	Pr(> |*t*|)
Intercept	107.2652	21.3819	5.017	2.42e‐05
Tmed_PreLP	−7.3715	3.0391	−2.426	0.0217
Tmed_PreLP^2^	0.2275	0.1073	2.120	0.426

Another issue in climate change research is whether climate variables directly influence breeding phenology (Sanz et al. 2003; Öberg et al. 2014). Long before climate change became an issue in long‐term population studies, associations between climate variables and breeding dates were a focus of long‐term population studies (Lack 1950; Perrins 1965). Some studies found that prelaying climatic conditions could directly influence egg‐laying date, with higher temperatures and lower precipitation inducing advancements in laying in some populations (Crick et al. 1997; McCleery and Perrins 1998; Sanz 2002; Goodenough et al. 2010; Källander et al. 2017; Shave et al. 2019), while others showed no such responses (Bailey et al. 2022). Increasing the number of years included in such studies increases the range of prelaying conditions experienced by populations and may reveal trends that a shorter number of years may fail to show (White 2019). Here we report two different results: in the reduced data set, warmer years are associated with earlier laying dates. This is an expected association as warmer spring temperatures can trigger earlier nesting behavior in birds (Charmantier and Gienapp 2014; Burgess et al. 2018). This trend is only significant when including more years with unusually low prelaying temperatures (10°C). However, the nonlinear association for the three decades reveals a more complex scenario. While the overall trend shows that higher temperatures are associated with earlier breeding, conditions of prelaying temperatures above 15°C lead to delays in laying. Only a long sequence of years may include such rare conditions as prelaying temperatures above 15°C and reveal breeding constraints not often considered in long‐term studies. How hot temperatures operate proximally in delaying laying is not known but may involve fast pupation of caterpillars. Early cold snaps have been shown to lead to breeding failure in the population (Moreno et al. 2015), but early heat events may also interact negatively with flycatcher breeding processes and delay phenology. Precipitation before laying has also been related to the breeding date (Hidalgo Aranzamendi et al. 2019; Cox et al. 2019; Skwarska et al. 2022). However, in our study, prelaying precipitation does not seem to affect breeding phenology when including further years.

### 3.2 Nestling Condition

#### 3.2.1 Temporal Trend (Time‐Condition)

**FIGURE 4:**
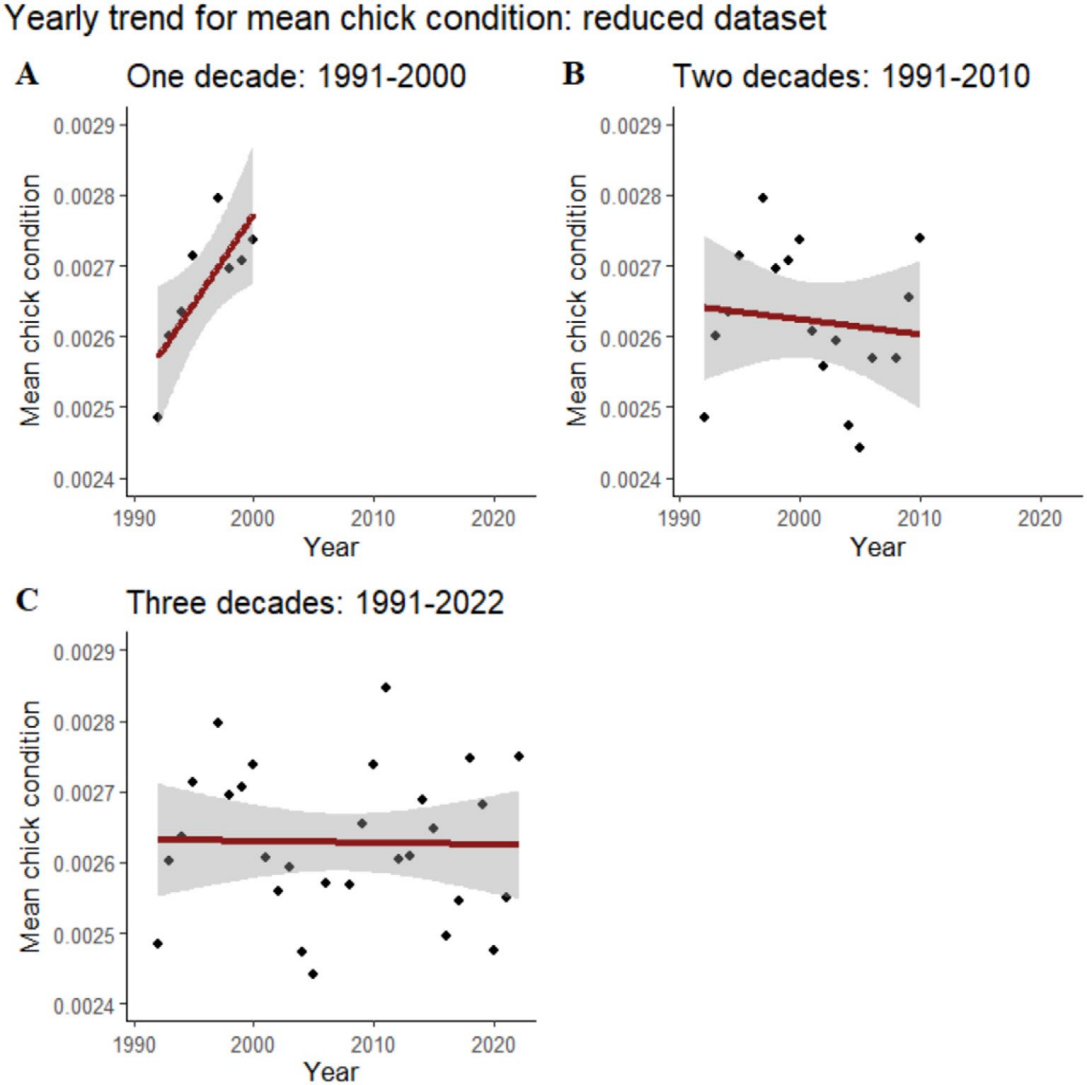
Temporal trends for nestling condition for the different study durations. (A) 1991–2000 (linear regression: *Y* = −0.05 + 0 x; *p* = 0.02), (B) 1991–2010 (linear regression: *Y* = 0.01 + 0 x; *p* = 0.32), and (C) 1991–2022 (linear regression: *Y* = 0 + 0 x; *p* = 0.9).

Based on AICc model selection, we selected linear models for our analyses. Only the first decade resulted in a significant model (Table 3); here we observed a rising trend delineating a better chick condition as years passed. As we included the second and third decades, the function loses its significance and becomes flat, showing no clear trend in condition across the three decades (Figure 4).

**TABLE 3:** Temporal linear model (Cc ~ Year) for 1991–2000.

Coefficients	Estimate	Std. error	*t* value	Pr(> |*t*|)
Intercept	−4.819e‐02	1.678e‐02	−2.872	0.0284
Year	2.548e‐05	8.408e‐06	3.031	0.0231

#### 3.2.2 Climatic Trends

The AICc model comparison indicated that, for all three study durations, the linear model provides a better explanation for the trend. Nevertheless, in all three cases, the estimates were found to be non‐significant, which aligned with the results of the full data set. For precipitation, linear functions again showed a better fit based on AICc model selection. However, linear functions did not explain changes in nestling condition.

The condition of nestlings has been used as a relatively accurate index of reproductive success in the context of climate change research (Mainwaring and Hartley 2016; Riggio et al. 2022). Some studies have shown declining trends across decades (Both et al. 2006; González‐Braojos et al. 2017), indicating negative climate impacts on breeding success. Nestling condition depends on the adjustment of breeding phenology to fluctuations in resource supply, so it is affected by temperature increases across the breeding season (Burger et al. 2012). A lack of adjustment may thus lead to decrements in nestlings' condition, resulting in declining rates of recruitment and consequent population declines (Both et al. 2006, 2009). Here we show that a short‐term improvement in nestling condition indicated in the first decade of the study did not survive further years of study. This temporal tendency to flatten out short‐term fluctuations may suggest that less than 3 decades (amounting to roughly ten generations in the species) is an insufficient period to extract conclusions about temporal trends. The absence of any association between climatic conditions during the nestling period and nestlings' condition across three decades of study strengthens the conclusion that temporal trends in climatic conditions are unable to affect this crucial breeding parameter in the study population.

### 3.3 Female Condition

#### 3.3.1 Temporal Trend (Time‐Condition)

**FIGURE 5:**
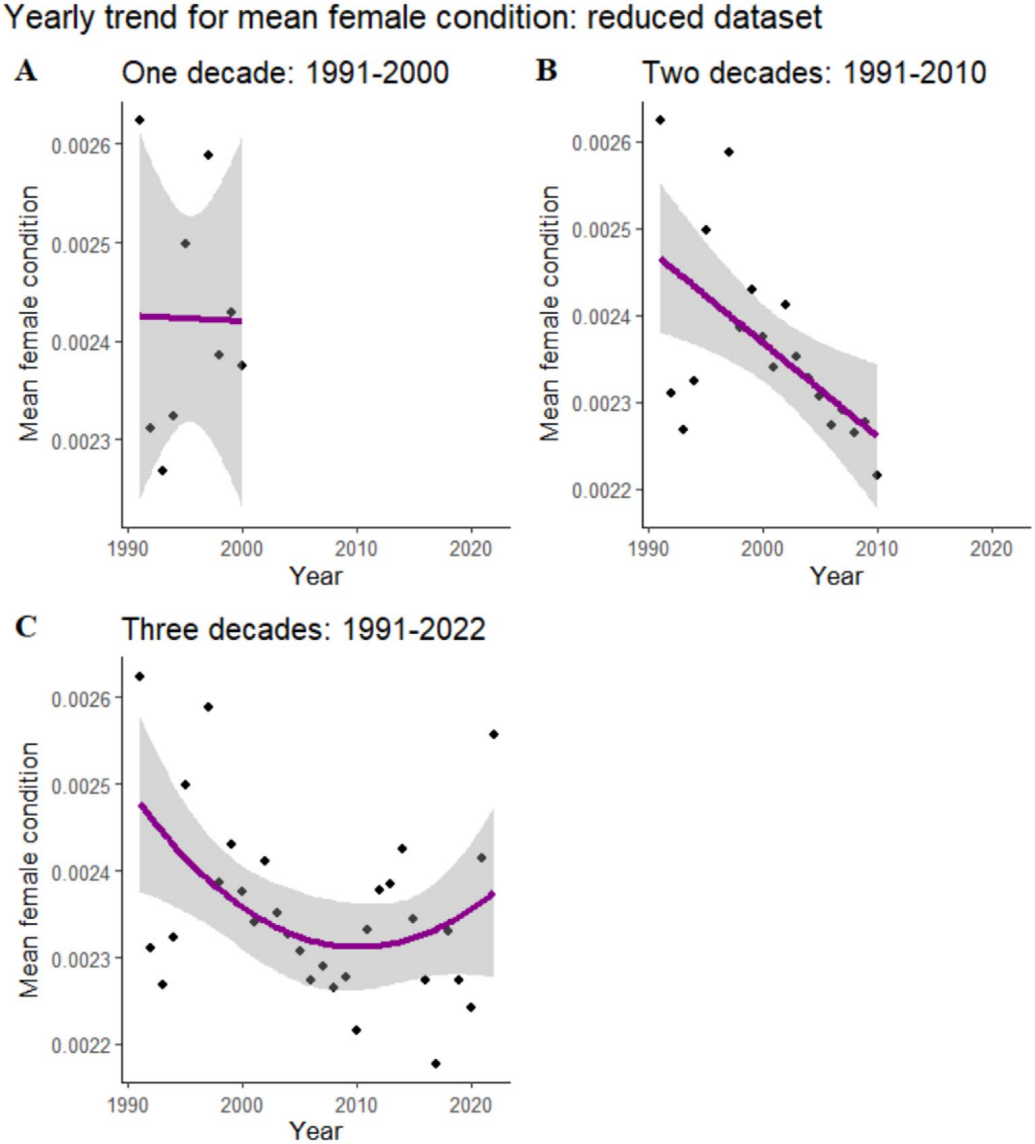
Temporal trends for female conditions for the different decades. (A) 1991–2000 (linear regression: Y = 0 + 0 x; *p* = 0.9), (B) 1991–2010 (linear regression: Y = 0.02 + 0 x; *p* = 0.007), and (C) 1991–2022 (quadratic regression: Y = 1.83 + 0 x1 + 0 x2; *p* = 0.04).

The linear model provided a better explanation of the yearly variation in female condition for both the first (1991–2000) and second (1991–2020) study durations. However, when considering the full three‐decade length of the study, the quadratic model outperforms the linear one. For the first decade, the model was non‐significant, but when adding the second decade, a significant negative trend was found: as the years passed, the female condition worsened (Table 4). Moreover, when considering the three‐decade period, we detect an overall decline in female condition, with the rate of decline in female condition slowing down with time (Figure 5).

**TABLE 4:** Linear and quadratic temporal models (Fem_cond ~ Year2) for (a) 1991–2010, (b) 1991–2022.

Coefficients	Estimate	Std. error	*t* value	Pr(> |*t*|)
(a) 1991–2010 (linear)				
Intercept	2.388e‐02	7.149e‐03	3.341	0.00388
Year	−1.076e‐05	3.573e‐06	−3.010	0.00788
(b) 1991–2022 (quadratic)				
Intercept	1.826e+00	9.006e‐01	2.027	0.0522
Year	−1.814e‐03	8.977e‐04	−2.021	0.0529
Year2	4.513e‐07	2.237e‐07	2.018	0.0533

#### 3.3.2 Climatic Trends

The AICc model comparison indicated that, for all three decades, linear functions for temperature during incubation better described variation in female condition. However, all three models were found to be non‐significant, aligning with the results of the full data set. Similar to the temperature analysis, linear models were the best ones, but trends became barely detectable when considering the three decades. In any case, neither the linear nor the quadratic functions for precipitation represented the variation in female condition, as found in the analysis with the full data set.

Female condition may affect reproductive success through the capacity for parental care (McRae 1998; Mock 2022) and is in itself an index of reproductive costs and post‐breeding survival prospects (Wesołowski 2004). Female condition declines have been related to negative population trends (Gardner et al. 2016) and may indicate maladaptation in the population to present environmental changes (Kouba et al. 2021). Here we show that only including data from the second decade revealed a significant trend in female condition. Interestingly, the initial negative trend detected for the two‐decade period is followed by an improvement in the last decade.

## 4 Conclusions

Prolonging our long‐term population study from one to two and then to three decades reveals a changing dynamic. Overall, the analysis shows that the absence of trends initially turns into significant tendencies when including further years (advancement in breeding dates only after more than two decades) or that some initial trends in reproductive variables change to their opposite (initial temporal increases in chick and female condition that turn into declines with further years). Including a greater range of conditions by prolonging the study may show unexpected results (revealing an advancement in laying when including low prelaying temperatures or delays in laying with very hot prelaying conditions). Also, some initial trends (nestling condition) vanish after further years of study, indicating adaptation of the population to climate‐driven changes. Declared tendencies in population parameters should be based on studies of three or more decades of duration for short‐lived passerines (ten or more generations) to have predictive value, since restricting the study duration poses limitations for an evaluation of the impact of global change on biodiversity (White 2019; Orgeret et al. 2021).

## Author Contributions

**Irene Zanandrea:** data curation (equal), formal analysis (equal), methodology (equal). **Juan Moreno:** conceptualization (equal), project administration (equal), resources (equal), writing – review and editing (equal). **Alejandro Cantarero:** conceptualization (equal), funding acquisition (equal), methodology (equal), project administration (equal), supervision (equal), writing – review and editing (equal).

## Ethics Statement

The study did not require ethical approval. The Junta de Castilla and León authorised the ringing and handling of birds. Work in the field area was done with permission from J. Donés and J. García Gámez (Director of “Montes de Valsain”).

## Conflicts of Interest

The authors declare no conflicts of interest.

## Data Availability

Data available from the Digital CSIC Repository: https://doi.org/10.20350/digitalCSIC/16994.
